# Parasites opt for the best of both worlds

**DOI:** 10.7554/eLife.49615

**Published:** 2019-08-01

**Authors:** Ellen Decaestecker, Lore Bulteel

**Affiliations:** Department of BiologyKatholieke Universiteit Leuven KulakKortrijkBelgium

**Keywords:** evolution of sex, outcrossing, epidemiology, *Plantago lanceolata*, *Podosphaera plantaginis*, coinfection, Other

## Abstract

The fungal parasite *Podosphaera plantaginis* employs both sexual and asexual reproduction to increase its chances of infecting the plant *Plantago lanceolata*.

**Related research article** Laine A-L, Barrès B, Numminen E, Siren JP. 2019. Variable opportunities for outcrossing result in hotspots of novel genetic variation in a pathogen metapopulation. *eLife* **8**:e47091. doi: 10.7554/eLife.47091

Most populations of animals produce genetically different offspring via sexual reproduction. A small minority of animals, however, use asexual reproduction to produce offspring that are genetically identical to the parent. Both strategies have their advantages and weaknesses, and some animals actually alternate between sexual and asexual reproduction (Decaestecker et al., 2009).

Most parasites reproduce asexually, but they can switch to sexual reproduction to encourage diversity and to remain infectious. Certain species of parasites can even sexually reproduce with other species, via a process called hybridization. For example, some schistosome flatworms can infect a wider range of hosts, as a result of a bovine-infecting species mating with a human-infecting species (Huyse et al., 2009). This demonstrates how variation caused by hybridization can increase the spread of a disease.

Another parasite that reproduces both sexually and asexually is the fungus *Podosphaera plantaginis*, which commonly infects the plant species *Plantago lanceolata*, commonly known as ribwort plantain (Figure 1). These fungi asexually reproduce by producing infective spores throughout their life-cycle. When the host’s growing season comes to an end *P. plantaginis* produce resting spores that can survive the winter. These spores can either be produced via mating, or via an asexual process known as haploid selfing that involves spores from the same organism 'mating' with each other (Tollenaere and Laine, 2013; Figure 1). Reproducing sexually, however, comes at a much greater cost, bringing into question why pathogens continue to maintain this reproductive strategy.

**Figure 1.**
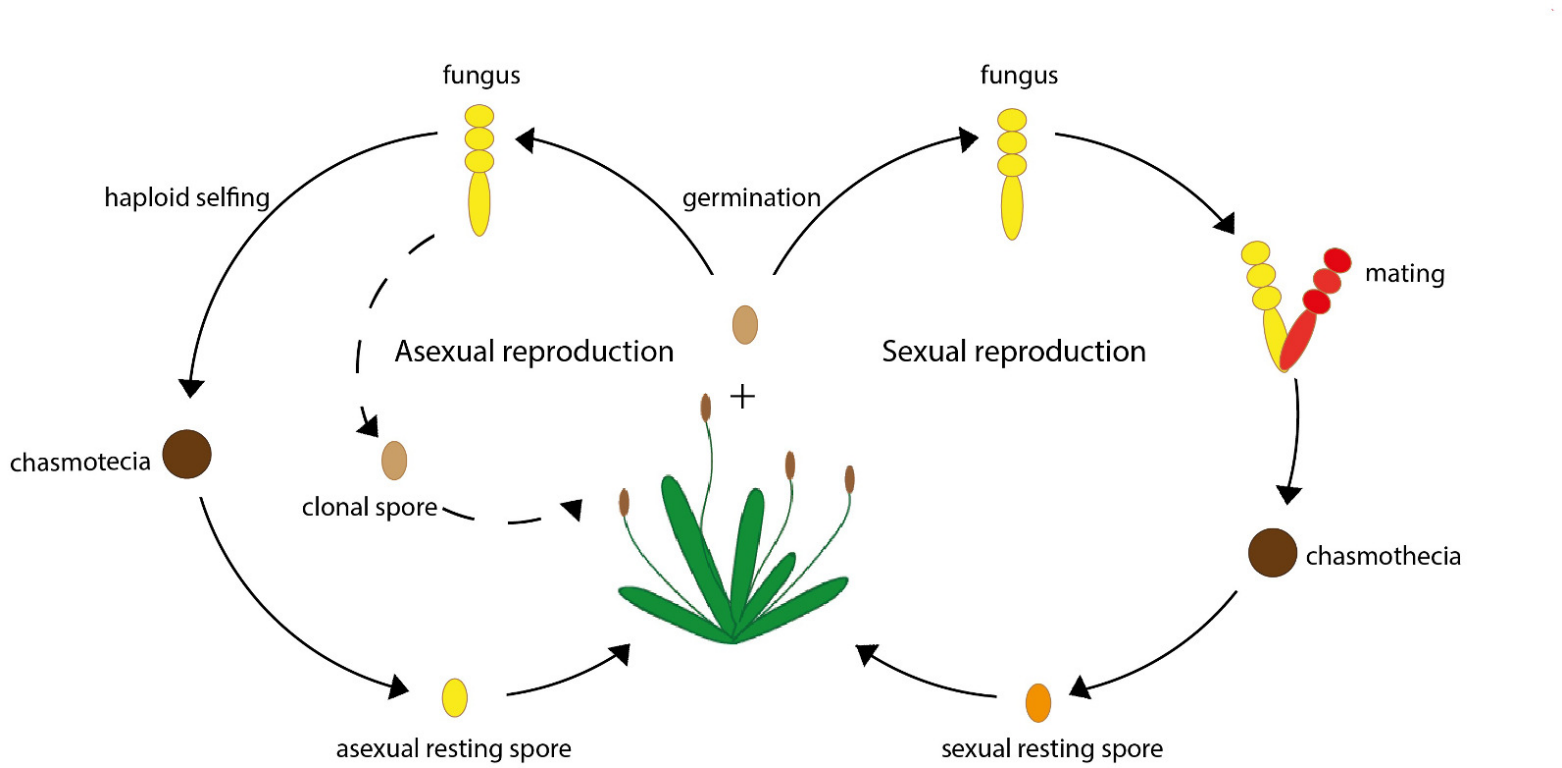
A simplified life cycle of the fungus *P. plantaginis.* The fungus *P. plantaginis* can reproduce both asexually (left) or sexually (right). Throughout the growing season *P. plantaginis* infects its host by asexually reproducing genetically identical clonal spores (represented by a dashed line). As the growing season of the host plant comes to an end, *P. plantaginis* produces a fruiting body known as chasmothecia, which contains resting spores that can survive the winter: these can either be reproduced asexually via process known as haploid selfing (left) or sexually via mating (right). When the next growing season starts, parasites can re-emerge from these resting spores and infect the host. Image credit: Lore Bulteel (CC BY 4.0).

One possible explanation comes from an evolutionary theory known as the Red Queen hypothesis. Hosts and parasite species constantly evolve together in order to outcompete one another: hosts co-evolve an increased resistance, whilst parasites increase their ability to infect. The Red Queen hypothesis proposes that hosts with a co-evolving parasite are more likely to reproduce sexually to increase genetic variation in their offspring so they have a greater chance of evading infection. Parasites can then evolve quickly and continuously in response to the ongoing evolution of the host’s defenses. This host-parasite arms race therefore offers an evolutionary advantage by helping maintain genetic variation in the population (Koskella and Lively, 2009; Decaestecker and King, 2019).

The time lag in co-evolution between these two organisms largely depends on the evolutionary potential (ability to genetically adapt in response to environmental pressures) of the parasite (Gandon et al., 2008). However, much of the evidence supporting the Red Queen hypothesis has come from host populations, and the evolutionary and ecological benefit of sexual reproduction in parasites has scarcely been described. Now in eLife, Jukka P Sirén of Aalto University and co-workers at the University of Helsinki, University of Zurich and Université Lyon – including Anna-Liisa Laine as first author – report evidence of sexual reproduction being maintained in subpopulations of the fungal parasite *P. plantaginis* (Laine et al., 2019).

Laine et al. tracked the life-cycle of *P. plantaginis* subpopulations in the Åland Islands of Finland over four consecutive years. Analyzing the DNA sequences of every *P. plantaginis* specimen revealed a number of new genotypes were being produced each year, suggesting that sexual reproduction is common within this fungal species. For sexual reproduction to occur at least two genotypically different parasites need to infect the same host so they can be close enough to exchange genetic material. Laine et al. showed the number of co-infected specimens was spatially varied across the population. *P. plantaginis* with an increased number of co-infections were also found to have a larger number of new genotypes, suggesting that there are localized hotspots of genetic diversity.

Further work showed that these populations with a higher prevalence of co-infections (and thus sexual reproduction) were also more likely to survive winter and initiate an infection the following season. This higher success indicates that these newly generated genotypes can start populations that are better equipped at infecting the host plant. When the growing season starts, this in turn could have an effect on the host population, and cause them to adapt a higher resistance.

Sub-populations of hosts and parasites will co-evolve at different rates depending on their surrounding environment: for example, in one location the parasite may be ahead in the evolutionary arms race, while in another the host is winning. These differences in evolutionary rate are then reflected spatially, creating localized pockets of emerging new genotypes (Thompson, 2005). By increasing genetic diversities in these areas, parasites can ‘hedge their evolutionary bets’ and deliver rare genotypes, which have lower fitness but could potentially thrive under certain stressful environmental conditions (Starrfelt and Kokko, 2012). It would therefore be interesting to explore how changes in selection pressures between *P. plantaginis* and its host effects the number of resting spores that are sexually produced. This could be achieved by setting up a bank of *P. plantaginis* resting spores, and tracking their infections throughout the years to see how they relate to evolutionary changes (Gaba and Ebert, 2009).

Future challenges involve seeing if the results obtained from this study can be reproduced by laboratory experiments, and untangling the possible selective pressures and benefits of sexual reproduction in parasites. It is also possible that sexually generated genetic changes in parasites could influence important ecological processes: for example, genetic variation in parasites could make a change to the host’s shape, which then propagates throughout the population and disrupts the whole ecosystem. These changes in the ecosystem could then fuel evolutionary changes in the parasite, resulting in a full feedback loop of eco-coevolutionary interactions.
